# Supplementary material for: Arsenic-Containing Medicine Treatment Disturbed the Human Intestinal Microbial Flora
Source: Toxics. 2023 May 15;11(5):458. doi: 10.3390/toxics11050458 (PMC10223770; doi:10.3390/toxics11050458)
Supplement: Supplementary file 1 [file toxics-11-00458-s001.zip › toxics-2368952-supplementary.pdf]

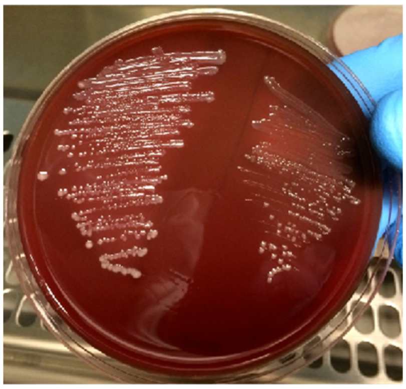

Figure S1. *B. fragilis* str. 3783N1-6 cells were initially grown on 1.5% solid agar plates.

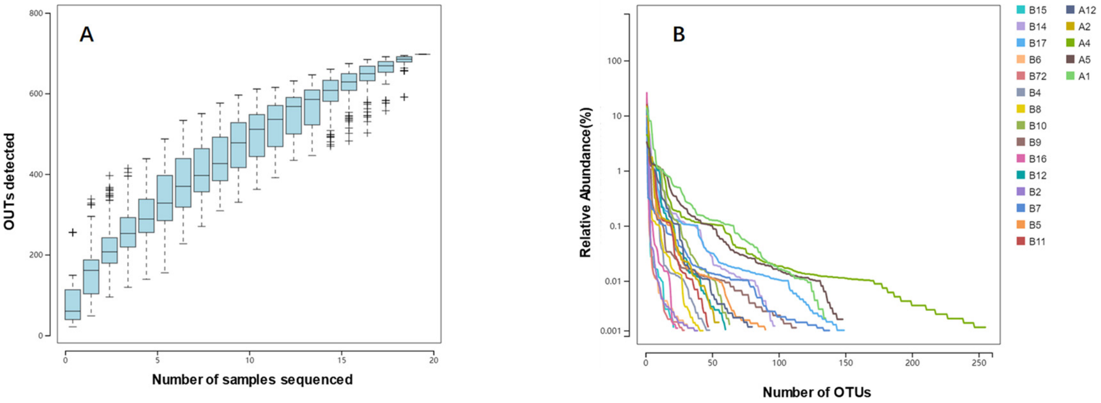

Figure S2. (**A**) Species accumulation curves. The x-axis showed number of samples sequenced, and the vertical coordinate represents OTU numbers. (**B**) OTURank curves with the OTU grade as the y-axis and the relative abundance of OTU as the x-axis.

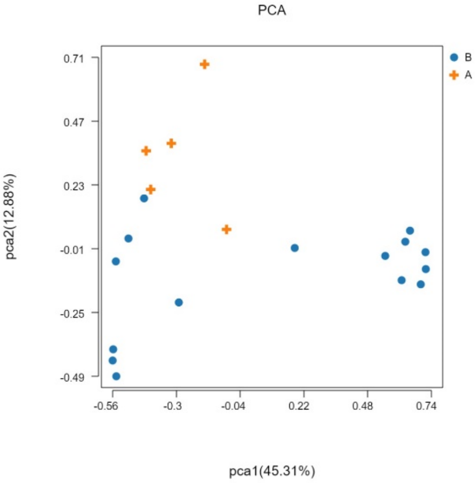

Figure S3. Microbial clustering visualized by principal coordinate analysis for the ATO group vs. control. PC1 can be considered as the main factor leading to the separation of the two groups. Points in different colors in the figure indicate that they come from different samples.

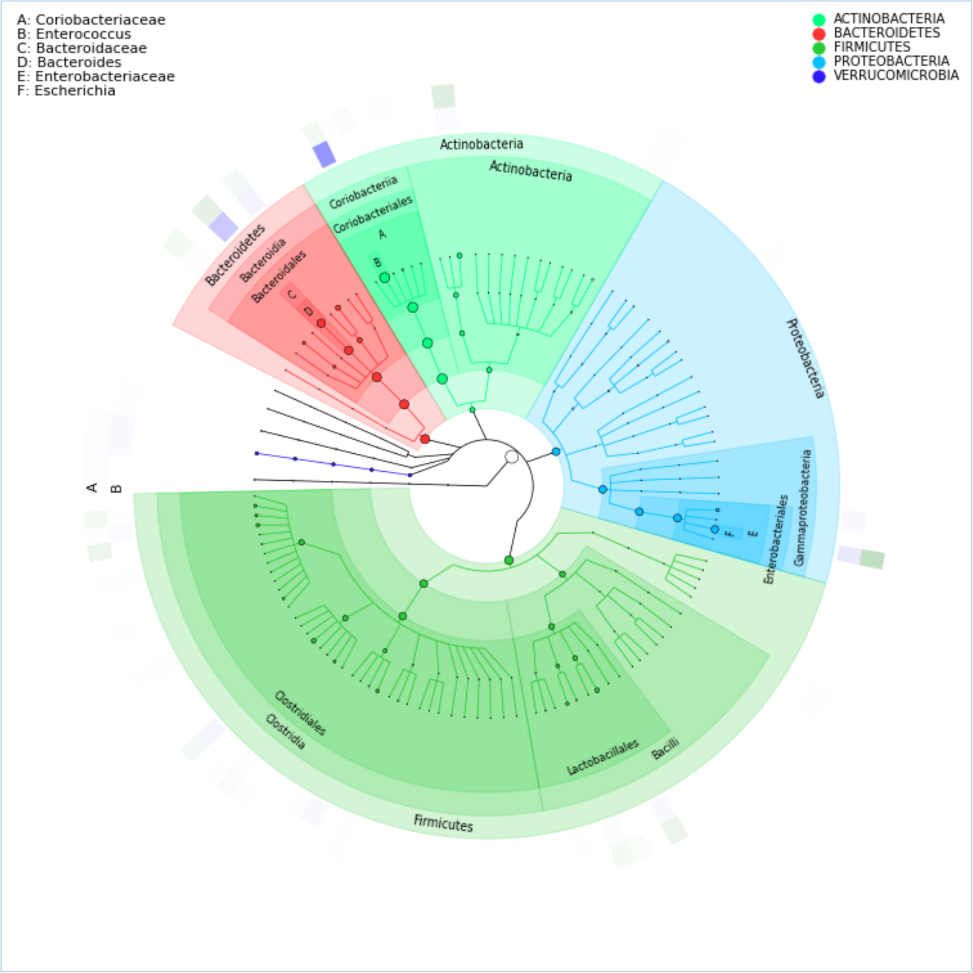

Figure S4. The taxonomy tree visualized by GraPhlAn.

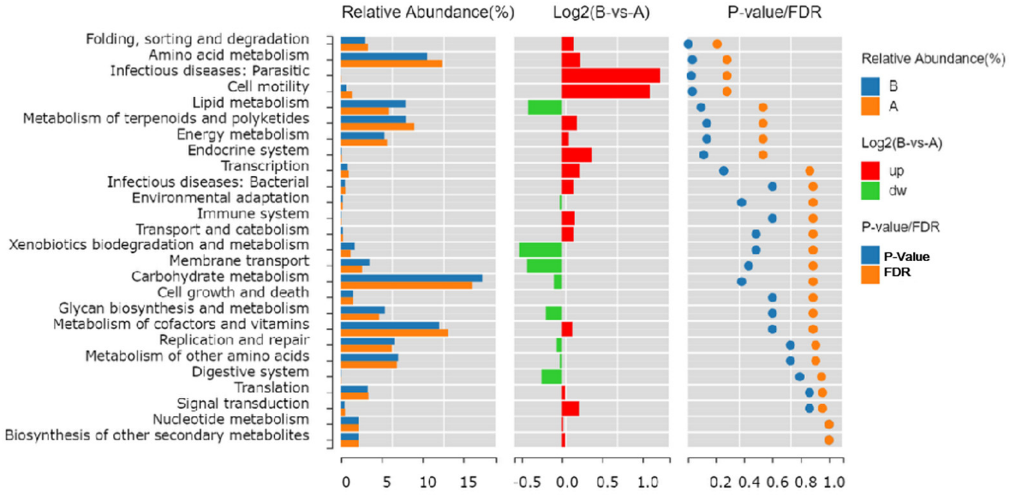

Figure S5. Functional difference in KEGG pathway pre - and post-AATO treatment.

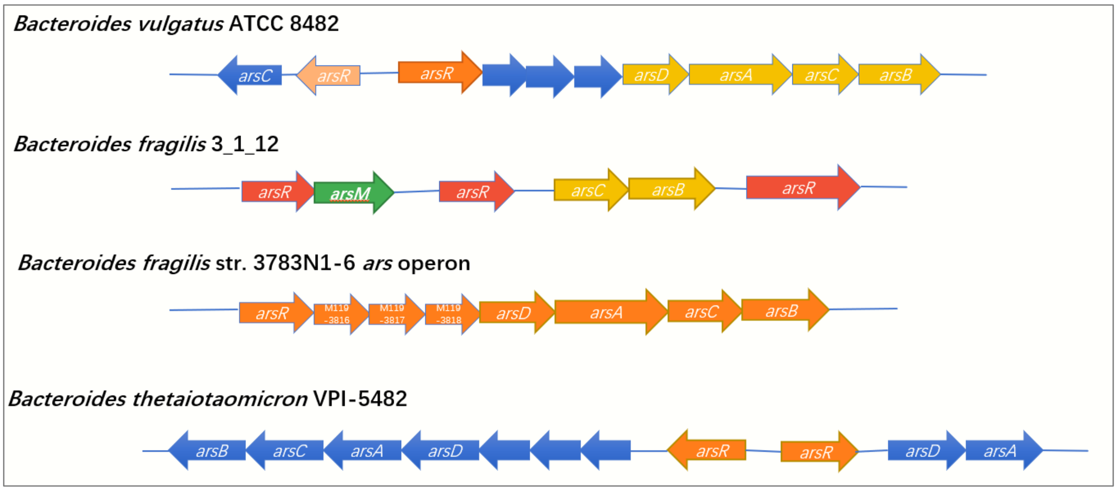

Figure S6. The *ars* operon in the genome of four Bacteroides from NCBI databank.

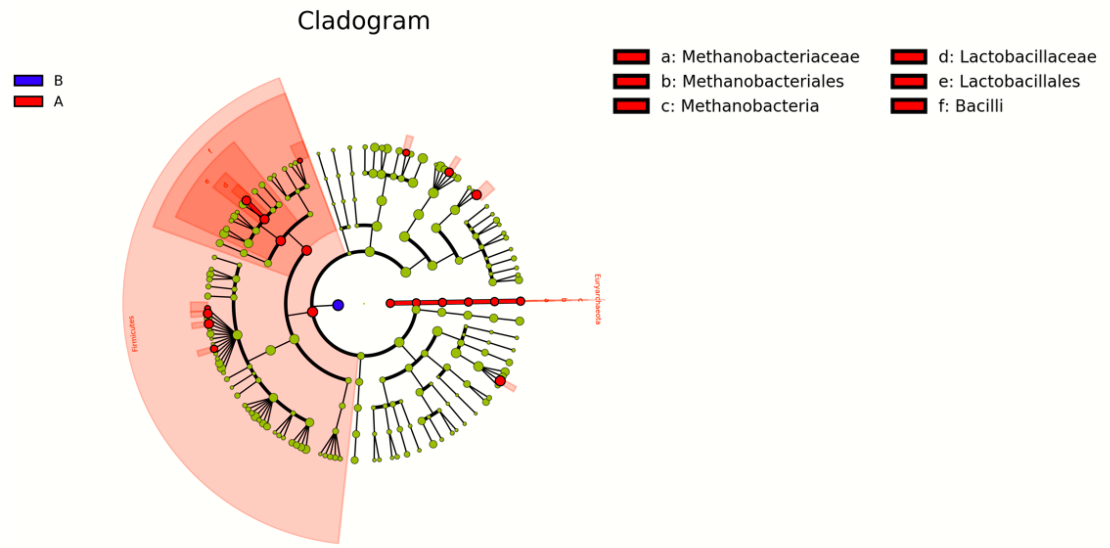

Figure S7. Cladogram for taxonomic representation of significantly difference among groups using the LefSe tool.

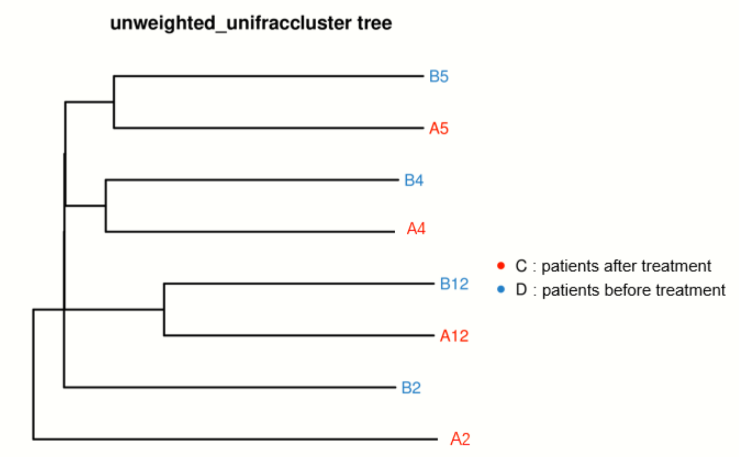

Figure S8. Unweighted-Unifrac cluster distance matrix for UPGMA clustering analysis of sample composition and differences. The clustering results were integrated with the relative species abundance of each sample at phylum, class, order, family, genus and species level. Software used: PhyTools, R(V3.5.1).

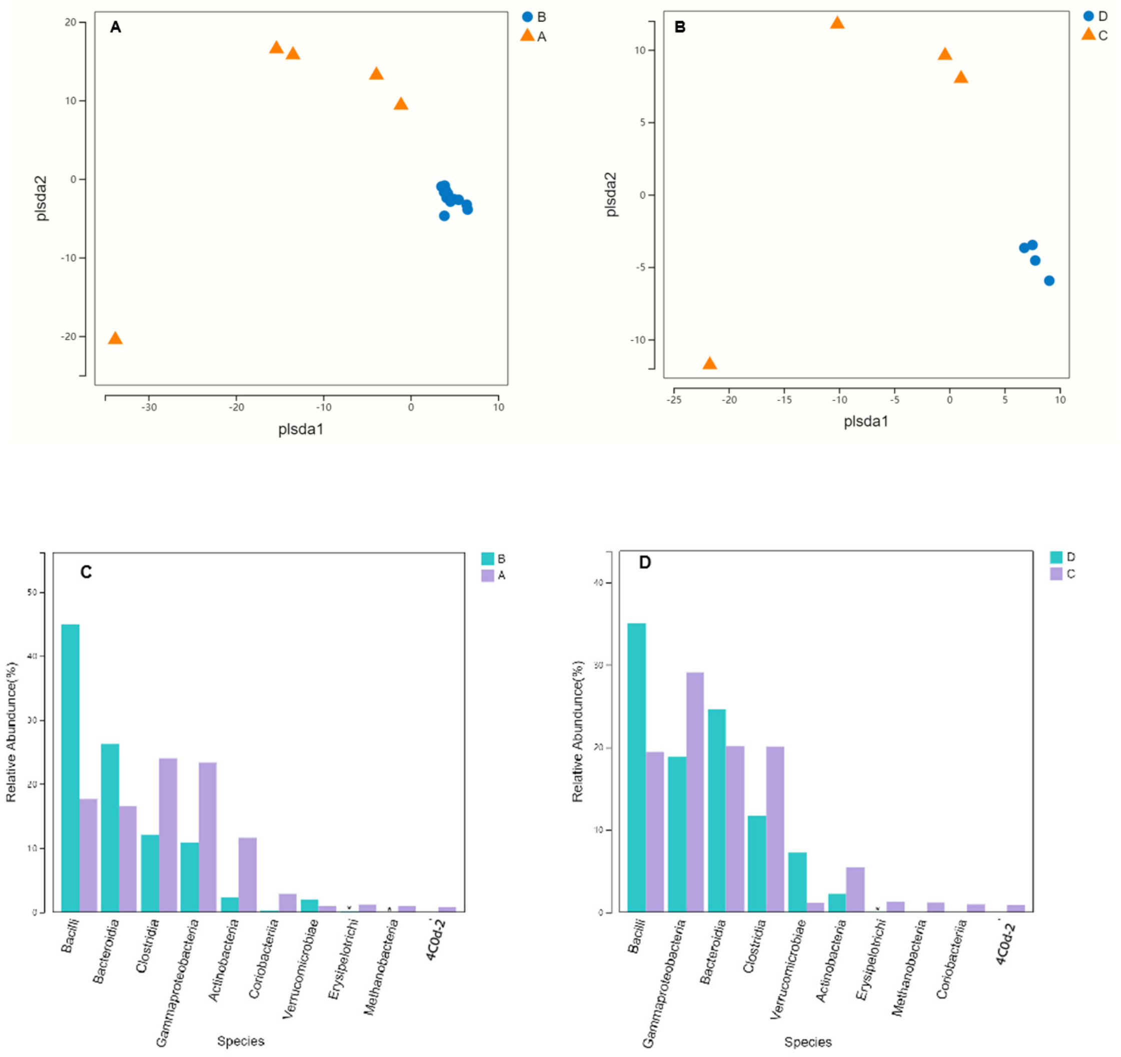

Figure S9. Comparison of AB and CD groups in Partial least squares discrimination analysis (PLS-DA) and the significant difference species. **(A)** PLS-DA of group A and B; (B) PLS-DA of group C and D; (C) The class level of key species of AB group. (D) The class level of key species of CD group. The scale on the horizontal and vertical axes is relative distance and has no practical significance. Use software: R(V3.2.1) mixOmics package.

Table S1. Oligonucleotide primers for qPCR. Expression of each gene was determined using the indicated qPCR primers.

Q-RT-PCR primer set	Target	Primer sequences
1	*arsA*	Sense: 5′- GAAAGGGTGGCGTAGGTAAA -3′<br>Antisense: 5′- GGGTCAGTGGTGGTTAGATG -3′
2	*acr3*	Sense: 5′- GCAAACGTCCGAGAGGAATAA -3′<br>Antisense: 5′- AGCTATCCCGAACATGGTAAAC -3′
3	*arsC*	Sense: 5′- CATCCGTTGGTCCTGAAACTAA -3′<br>Antisense: 5′- CGTACCGGAAAGTACGATGATATG -3′
4	*arsD*	Sense: 5′- ACGTAAGCAATAAGACGGTGAA -3′<br>Antisense: 5′- TTGGTGGTAGGATAGGTTTGTG -3′
5	16s rRNA	Sense: 5′- AACTGAGACACGGTCCAAAC -3′<br>Antisense: 5′- CCATAGAGCCTTCATCCTTCAC -3

Table S2. Table of Alpha diversity indices of intestinal microorganisms of APL patients before (A group) and after (B group) AATO treatment.

	**A group**	**B group**	***p*-value**
Chao1	134.60 ± 77.45	134.60 ± 77.45	0.053
Shannon	2.88 ± 0.85	1.49 ± 0.95	0.019
Simpson	0.14 ± 0.11	0.45 ± 0.29	0.025

Table S3. The taxonomic assignments and fold changes of gut bacterial components at family level that were significantly changed ($p < 0.05$, marked by yellow color).

**Bacteria**	**A(%)**	**B(%)**	***p*-value**	**FDR**
Lactobacillaceae	5.42096	0.951826	0.008327	0.296967
Erysipelotrichaceae	1.162445	0.127933	0.01124	0.296967
Methanobacteriaceae	0.940536	0.0	0.015126	0.296967
Peptostreptococcaceae	2.633587	0.120485	0.017998	0.296967
Clostridiaceae	0.841942	0.275201	0.02798	0.369336
Coriobacteriaceae	2.814256	0.233476	0.054453	0.58283
Bifidobacteriaceae	11.23595	1.708333	0.066388	0.58283
Alcaligenaceae	0.013559	0.573839	0.090168	0.58283
Enterobacteriaceae	23.223007	10.798081	0.0971	0.58283
Victivallaceae	6.32E-4	0.0	0.105969	0.58283
Dehalobacteriaceae	0.007903	0.0	0.105969	0.58283
Listeriaceae	0.00364	0.0	0.105969	0.58283
Rikenellaceae	0.533709	0.178358	0.136753	0.585774
Turicibacteraceae	0.300631	0.004093	0.151037	0.585774
Pasteurellaceae	0.143838	0.004434	0.151037	0.585774
Bacillaceae	0.006259	0.004163	0.161815	0.585774
Lachnospiraceae	12.413791	3.808643	0.161903	0.585774
Ruminococcaceae	6.515893	4.451026	0.162536	0.585774
Aerococcaceae	0.0	0.046551	0.168632	0.585774
Enterococcaceae	4.116168	41.166538	0.19043	0.600828
Barnesiellaceae	0.04702	0.041707	0.196573	0.600828
Prevotellaceae	3.717521	0.552019	0.200276	0.600828
Corynebacteriaceae	0.0	0.011136	0.235559	0.675952
Odoribacteraceae	0.099786	0.047032	0.28348	0.728642

Table S4. Arsenic concentration in selected urine and feces samples.

**Random Selected Sample**	**Sample Types**	**Results**	**OTUs**
B1	urine	0.75 mg/L	——
B4	urine	0.39 mg/L	48
B5	urine	0.32 mg/L	90
B6	urine	0.43 mg/L	35

A2	urine	ND	55
B8	urine	0.38 mg/L	43
B9	urine	1.33 mg/L	113
B2	Feces	0.33 mg/kg	39
B4	Feces	0.06 mg/kg	48
B7	Feces	0.02 mg/kg	138
B12	Feces	0.20 mg/kg	60
B17	feces	1.03 mg/kg	149
